# That Banquet

**Published:** 1894-05

**Authors:** 


THAT BANQUET.—The banquet given by the Austin physicians to the Texas State Medical Association was a most humiliating failure; there was not enough to eat, and what there was, was inferior; the celery stale, the fruit rotten, and the wine indifferent. Dr. Bennett, chairman of the Committee of Reception, made an explanation the last day of the session, placing the blame where in belongs; but as there were very few present, the committee stands in a bad light before the profession, and is no doubt laughed at, or otherwise unjustly criticised. We therefore make the same explanation here, for the benefit of those who did not hear it.

There was an abundance of money, and an abundance of good will and good intention and liberality. The committee had raised from the local profession several hundred dollars more than the contract price of the banquet (ordered for 225) came to; they had money to spare. So the scant supper was in no way due to scant funds, nor stinginess. The committee procured a *menu* from each of several caterers here in Austin, with their bid to furnish supper and wine for 225. The management of the brag "Driskill" submitted the most satisfactory *menu*, and said to Dr. Bennett, in effect, "We want the order, and will do better than any one *can* do; we will *spread* ourselves, and will guarantee satisfaction, and will set a supper that will be a credit to you and to us; we do not expect to make a cent, but want to set the supper as an advertisement for the hotel."

Well, they got the advertisement, and much good may it do them, if it is any satisfaction to them that at least seventy-five physicians and ladies sat down to empty tables and left without gettiug a mouthful of anything to eat; while the one hundred who entered first cleaned up the supper and left, dissatisfied, and criticising the whole affair. There was not food enough for one hundred, and it was all put on the table at one time, and those who entered first ate it all. There was $230 left, after paying a round price per head for all who entered, including those who left without getting anything to eat; and all other bills.

The custom of giving a banquet ought to be abolished. True, it is not obligatory to set a supper, but the precedent has been observed up to date by every committee where the Association has met, for several years, and with each succeeding committee it appears to be a case of *noblesse oblige.* It is a tax upon the local profession wherever the Association meets. With all other conventions of whatever kind, religious, railroad, cattle men, or political, whenever a banquet is given, the business men and citizens generally are called upon for funds; but when the doctors meet the local brethren bear all the burden. And yet every attempt made in Association meetings to vote down the custom is defeated. One result of the custom is—nobody wants the Convention. At this meeting, Dr. Blailock offered a resolution to the effect that in future the brethren where the meeting is to take place "shall not feel *obliged by precedent* to set out a banquet," and it was promptly voted down. Dallas, where we meet next (without an invitation, however), will please take notice. The defeat of the resolution is, in effect, to say that hereafter, wherever the Association elects to meet, the profession *must feel obliged by precedent to set a banquet!*

The fact is, these Conventions should be held wherever there are best accommodations, and at most accessible points; should hire their own hall, and each man go to a hotel and pay his expenses, and the local profession should not be put to expense of and kind for their entertainment.

---

IN THE election of Dr. McLaughlin to the Presidency, the Texas State Medical Association honored itself. Dr. McLaughlin is a man of dignified presence, and is a popular, courteous physician. He is better known in connection with original scientific research than any physician, perhaps, in Texas. His researches on the dengue microbe gave him even European reputation; while his writings on Infection, Contagion and Immunity have awakened a wide spread interest in medical circles throughout the world. He has entered a new field, and if he has really made no discovery he has at least blazed the way for others and opened up a wide field for investigation. We present herewith a picture of our new President, of which the JOURNAL is quite proud, and a brief biographical sketch of his life. In his younger days Doctor McLaughlin was a dashing cavalry captain in the Confederate service, and waged a semi-guerilla warfare in the mountains of Tennessee, under the dashing Morgan and Duke, making many narrow escapes. A part of his life if written out would read like a romance.

